# Supplementary material for: A Bayesian approach to reveal the key role of mask wearing in modulating projected interpersonal distance during the first COVID-19 outbreak
Source: PLoS One. 2021 Aug 10;16(8):e0255598. doi: 10.1371/journal.pone.0255598 (PMC8354471; doi:10.1371/journal.pone.0255598)
Supplement: S1 File — (DOCX) [file pone.0255598.s001.docx]

**Supplemental Information**

A Bayesian approach to reveal the key role of mask wearing in modulating projected interpersonal distance during the first COVID-19 outbreak

Matteo P. Lisi, Marina Scattolin, Martina Fusaro, and Salvatore Maria Aglioti

**Online Survey**

In the beginning of 2020 a disease, named COVID-19, started to spread in many Countries, infecting hundreds of thousands of people and dramatically causing many deaths. Some Countries have decided to impose strict restrictions to slow down the spread and contain the infections: for example, closing school and universities, suggesting social- distancing and, in some cases, imposing the quarantine and lockdown of the entire country.
The aim of the current survey is to collect data about how the virus is affecting our daily behaviour. Please, remember that there is no right or wrong answer. You will be asked to answer some general questions about yourself and your attitudes. Then, you will be asked to move a person on a line, imaging the distance you would keep between you and her/him to make you feel comfortable.

**Gender**

To which gender do you most identify?

1. Male
2. Female
3. Transgender F to M
4. Transgender M to F

**Sexual orientation**

1. Exclusively heterosexual
2. Predominantly heterosexual, only incidentally homosexual
3. Predominantly heterosexual, but more than incidentally homosexual
4. Equally heterosexual and homosexual
5. Predominantly homosexual, but more than incidentally heterosexual
6. Predominantly homosexual, only incidentally heterosexual
7. Exclusively homosexual
8. No socio-sexual contacts or reactions

**Ethnicity**

1. Hispanic or Latino or Spanish Origin of any race
2. American Indian or Alaskan Native
3. Asian
4. Native Hawaiian or Other Pacific Islander
5. Black or African American
6. Caucasian
7. Two or more races

**Age**

1. 18-24 years old
2. 25-34 years old
3. 35-44 years old
4. 45-54 years old
5. 55-64 years old
6. 65-74 years old
7. 75 years or older

**Education**

1. No schooling completed
2. Some high school, no diploma
3. High school graduate, diploma or the equivalent
4. Some college credit, no degree
5. Trade/technical/vocational training
6. Bachelor’s degree
7. Master’s degree
8. Professional degree
9. Doctorate degree

**Marital status**

1. Single, never married
2. In a relationship (not domestic partnership)
3. Married or domestic partnership
4. Widowed
5. Divorced
6. Separated

**Employment status**

1. Employed for wages
2. Self-employed
3. Out of work and looking for work
4. Out of work but not currently looking for work
5. A homemaker
6. A student
7. Military
8. Retired
9. Unable to work

**Political orientation**

How would you define yourself politically?

1. Very conservative
2. Conservative
3. Liberal
4. Very Liberal

**Religion**

What is your religion?

1. Catholic
2. Protestant
3. Ortodox
4. Jehovah's Witness
5. Buddhist
6. Hindu
7. Muslim
8. Jewish
9. Atheist
10. Agnostic
11. Other religion

**Nationality**
The following item allows the participant to enter information with the keyboard

**Country of Residence**

What State do you live in?
The following item allows the participant to enter information with the keyboard

**City of Residence**

What city (and district/county/region) do you live in?
The following item allows the participant to enter information with the keyboard

**Domicile**
In which city (and district/county/region) are you now?
The following item allows the participant to enter information with the keyboard

**Perceived severity of the situation in the country**

In your opinion, how serious is the situation related to COVID-19 in your country?
In this item, participants need use a slider to enter their information (0-100),

**Perceived severity of the situation in the world**

In your opinion, how serious is the situation related to COVID-19 in the entire world?
In this item, participants need use a slider to enter their information (0-100).

**Domestic partnership**

You are living:

1. alone
2. with your partner
3. with your family
4. with flatmates/ friends
5. others

**Virtual contact prior to the participation**

How often did you have VIRTUAL contacts (for instance, through Skype, Zoom, Whatsapp, etc) in the last two weeks?

1. Never
2. Rarely
3. Sometimes
4. Often
5. Always

**Physical contact prior to the participation**

How often did you have PHYSICAL contacts (for instance, hugs, cuddling, handshakes, etc) in the last two weeks?

1. Never
2. Rarely
3. Sometimes
4. Often
5. Always

**Altruism Scale**

Check the category on the right that conforms to the frequency with which you have carried out the following acts.
Each of the following lines (below) is rated using the following scale:

1. Never
2. Once
3. More than once
4. Often
5. Very often

The following lines need to be rated using the above scale:

1. I have helped push a stranger’s car out of the snow.
2. I have given directions to a stranger.
3. I have made change for a stranger.
4. I have given money to a charity.
5. I have given money to a stranger who needed it (or asked me for it).
6. I have donated goods or clothes to a charity.
7. I have done volunteer work for a charity.
8. I have donated blood.
9. I have helped carry a stranger’s belongings (books, parcels, etc.).
10. I have delayed an elevator and held the door open for a stranger.
11. I have allowed someone to go ahead of me in a lineup (at photocopy machine, in the supermarket).
12. I have given a stranger a lift in my car.
13. I have pointed out a clerk’s error (in a bank, at the supermarket) in undercharging me for an item.
14. I have let a neighbour whom I didn’t know too well borrow an item of some value to me (e.g., a dish, tools, etc.)
15. I have bought ‘charity” Christmas cards deliberately because I knew it was a good cause.
16. I have helped a classmate who I did not know that well with a homework assignment when my knowledge was greater than his or hers.
17. I have before being asked, voluntarily looked after a neighbour’s pets or children without being paid for it.
18. I have offered to help a handicapped or elderly stranger across a street.
19. I have offered my seat on a bus or train to a stranger who was standing.
20. I have helped an acquaintance to move households.

**Perceived Vulnerability to Disease**

Considering the current COVID-19 pandemic, how much do you agree with each of the following
Each of the following lines (below) is rated using the following scale:

1. Disagree strongly
2. Disagree moderately
3. Disagree a little
4. Neither agree nor disagree
5. Agree a little
6. Agree moderately
7. Agree strongly

The following lines need to be rated using the above scale:

1. In general, I am very susceptible to colds, flu and other infectious diseases.
2. I am unlikely to catch a cold, flu or other illness, even if it is ‘going around’
3. If an illness is ‘going around’, I will get it.
4. My immune system protects me from most illnesses that other people get.
5. I am more likely than the people around me to catch an infectious disease.
6. My past experiences make me believe I am not likely to get sick even when my friends are sick.
7. I have a history of susceptibility to infectious disease.
8. I prefer to wash my hands pretty soon after shaking someone’s hand.
9. I do not like to write with a pencil someone else has obviously chewed on.
10. I dislike wearing used clothes because you do not know what the last person who wore it was like.
11. I am comfortable sharing a water bottle with a friend.
12. It really bothers me when people sneeze without covering their mouths.
13. It does not make me anxious to be around sick people.
14. My hands do not feel dirty after touching money.

**Public Attitudes toward the Quarantine**

Considering the current COVID-19 pandemic, how much do you agree with each of the following
Each of the following lines (below) is rated using the following scale:

1. Disagree strongly
2. Somewhat disagree
3. Neutral
4. Somewhat agree
5. Strongly agree

The following lines need to be rated using the above scale:

1. Public Health should have the power to order people into Quarantine during outbreaks
2. Quarantine is a good way to stop the spread of infectious disease outbreaks
3. If someone is given a quarantine order by Public Health, they should follow it no matter what else is going on in their life at work or home
4. If I go into quarantine, my family/friends/community will be protected from becoming sick
5. People who break quarantine orders on purpose should face legal penalties like a fine or jail
6. Public Health should be able to lock people up if they fail to obey quarantine orders
7. Public Health should use electronic bracelets and in-home surveillance cameras for people who disobey quarantine orders
8. Answer "Somewhat agree"
9. Public Health needs to explain to everyone why they should be allowed to use quarantine
10. Government should pay for nurses and counselors to help people who are in quarantine
11. Public Health should ensure that people have food and shelter while in quarantine, and pay for it with public money if need be
12. Government should pay for counselors and support groups so that people coming out of quarantine have someone to talk to about it
13. People in quarantine should get money from the government to pay for missed time at work
14. Public Health should ensure that there is no discrimination in the use of quarantine
15. It is reasonable for some rights to be taken away during an infectious disease outbreak
16. People who disagree with their quarantine order should be able to request a review to have it ended early

**Moral Foundations Questionnaire**

When you decide whether something is right or wrong, to what extent are the following considerations relevant to your thinking? Please, considering the current COVID-19 pandemic, rate each statement using this scale:


not at all relevant

not very relevant

slightly relevant

somewhat relevant

very relevant

extremely relevant


Each of the following lines (below) is rated using the following scale:

1. not at all relevant
2. not very relevant
3. slightly relevant
4. somewhat relevant
5. very relevant
6. extremely relevant

The following lines need to be rated using the above scale:

1. Whether or not someone suffered emotionally
2. Whether or not some people were treated differently than others
3. Whether or not someone’s action showed love for his or her country
4. Whether or not someone showed a lack of respect for authority
5. Whether or not someone violated standards of purity and decency
6. Whether or not someone was good at math
7. Whether or not someone cared for someone weak or vulnerable
8. Whether or not someone acted unfairly
9. Whether or not someone did something to betray his or her group
10. Whether or not someone conformed to the traditions of society
11. Whether or not someone did something disgusting
12. Whether or not someone was cruel
13. Whether or not someone was denied his or her rights
14. Whether or not someone showed a lack of loyalty
15. Whether or not an action caused chaos or disorder
16. Whether or not someone acted in a way that God would approve of

    Please, considering the current COVID-19 pandemic, read the following sentences and indicate your agreement or disagreement
    Each of the following lines (below) is rated using the following scale:
17. Strongly disagree
18. Moderately disagree
19. Slightly disagree
20. Slightly agree
21. Moderately agree
22. Strongly agree

The following lines need to be rated using the above scale:

1. Compassion for those who are suffering is the most crucial virtue
2. When the government makes laws, the number one principle should be ensuring that everyone is treated fairly.
3. I am proud of my country’s history.
4. Respect for authority is something all children need to learn.
5. People should not do things that are disgusting, even if no one is harmed.
6. It is better to do good than to do bad.
7. One of the worst things a person could do is hurt a defenseless animal
8. Justice is the most important requirement for a society
9. People should be loyal to their family members, even when they have done something wrong.
10. Men and women each have different roles to play in society.
11. Answer "Moderately disagree"
12. I would call some acts wrong on the grounds that they are unnatural.
13. It can never be right to kill a human being.
14. I think it’s morally wrong that rich children inherit a lot of money while poor children inherit nothing.
15. It is more important to be a team player than to express oneself.
16. If I were a soldier and disagreed with my commanding officer’s orders, I would obey anyway because that is my duty
17. Chastity is an important and valuable virtue.

**Interpersonal Distance Visual Analogue Scale**

**Instructions**

Imagine that you are the person on the left of the line and that you cannot turn nor move. Then, imagine that the other person, depicted on the line, begins walking toward you. You should indicate how close you would allow this person to approach you while still being comfortable with that distance. To indicate where the other should stop, click on the horizontal line. Then, press “Next” to move to the following trial”.
During the task you will be approached by men and women, that may or may not be wearing masks and gloves. On the top center of the screen you'll read some information about the results of their Covid-19 test: a red sign reporting **"Covid-19 +"** indicates a person that tested positive; a green **"Covid-19 -"** indicates a person whose results were negative; a grey **"Covid-19 ?"** indicates that that person was not tested or that results are unknown.
