# Supplementary material for: A Bayesian approach to reveal the key role of mask wearing in modulating projected interpersonal distance during the first COVID-19 outbreak
Source: PLoS One. 2021 Aug 10;16(8):e0255598. doi: 10.1371/journal.pone.0255598 (PMC8354471; doi:10.1371/journal.pone.0255598)
Supplement: Attachment — Submitted filename: Response to Reviewers.docx [file pone.0255598.s003.docx]

^Sapienza University of Rome^

Department of Psychology

Subject: PONE-D-21-03526R1, revision

Rome, 16th July, 2021

Dear Prof. Capraro,

We wish to thank you for your professional handling of our manuscript entitled “**A Bayesian approach to reveal the key role of mask wearing in modulating projected interpersonal distance during the first COVID-19 outbreak**” (Authors Lisi, Scattolin, Fusaro, Aglioti). We wish to express our gratitude for the reviewer's comment and for all editorial suggestions, which we have taken into the highest consideration while revising our MS.

Following your recommendation, we enclose a copy of the revised MS with all the changes tracked, so that they can be easily identified. We also enclose a reply to the comment of the reviewer (our responses in bold; additions to the revised MS in bold&red).

We hope that you and the reviewers are satisfied with the revision and that the MS can now be accepted for publication in PLOSOne.

Sincerely yours,

Matteo P. Lisi and Salvatore Maria Aglioti (Correspondent Authors).

Sapienza University of Rome and Center for Life Nano- & Neuro-Science, Fondazione Istituto Italiano di Tecnologia (IIT), 00161 Rome, Italy

E-mail:

[matteo.lisi@uniroma1.it](mailto:matteo.lisi@uniroma1.it)

[salvatoremaria.aglioti@uniroma1.it](mailto:salvatoremaria.aglioti@uniroma1.it)

**Reviewer #2**

*The authors have adequately addressed my comments. This manuscript is now acceptable for publication.*

*I however recommand modifying the periaqueducal grey definition "(a defensive region...)" which is, in my opinion, a bit awkward.*

**ARRef2:**

**We thank the Reviewer for pointing out this.**

**We modified the sentence (page 27, lines 422-425), which now reads as follows: “Indeed, Vieira and colleagues [64] showed that distance from other organisms (conspecifics or not) is regulated by a network that includes the midbrain periaqueductal gray (a region sensitive to threat proximity and involved in defensive behaviors) and frontoparietal structures representing peripersonal space.”**

**--------------------------------------------------------------------------**
